# A core competency model for clinical informationists

**DOI:** 10.5195/jmla.2021.1065

**Published:** 2021-01-01

**Authors:** Mohammadreza Hashemian, Firoozeh Zare-Farashbandi, Nikoo Yamani, Alireza Rahimi, Peyman Adibi

**Affiliations:** 1 mr.hashemian553@gmail.com, Health Information Technology Research Center, Isfahan University of Medical Sciences, Isfahan, Iran; 2 f_zare@mng.mui.ac.ir, Health Information Technology Research Center, Isfahan University of Medical Sciences, Isfahan, Iran; 3 yamani@edc.mui.ac.ir, Medical Education Research Center, Isfahan University of Medical Sciences, Isfahan, Iran; 4 a_rahimi@mng.mui.ac.ir, Health Information Technology Research Center, Isfahan University of Medical Sciences, Isfahan, Iran; 5 adibi@med.mui.ac.ir, Integrative Functional Gastroenterology Research Center, Isfahan University of Medical Sciences, Isfahan, Iran

## Abstract

**Objectives:**

Access to high-quality information improves the quality of patient care, but lack of time and sufficient skills in information seeking can prevent access to information by clinicians. To solve this problem, clinical informationists can provide high-quality, filtered information for clinical team members. This study identified the core competencies that clinical informationists need to effectively fulfill their roles on clinical teams.

**Methods:**

Participants were selected purposefully from clinicians and medical librarians. Data were collected through semi-structured interviews and analyzed using qualitative content analysis.

**Results:**

The authors identified six competencies—communication, research, education and training, domain knowledge, information services, and technology—which together were used to develop a “CREDIT” model of core competencies for clinical informationists.

**Conclusions:**

The CREDIT model can be used as criteria for evaluating the performance of clinical informationists as well as for developing and assessing clinical informationist educational programs and curriculums.

## INTRODUCTION

Timely access to high-quality information by clinical team members is critical for the diagnosis and treatment of patients. The use of strong evidence reduces medical errors and ensures the quality of patient care [1]. However, factors such as lack of time and insufficient information-seeking skills prevent access to information [2, 3]. Because of these barriers, many clinicians and physicians prefer to get needed information from convenient and easy sources, which may not be accurate [4]. One way to overcome these barriers is to use clinical informationists (CIs), who have a thorough understanding of health care, information searches, and critical appraisal and who serve as members of clinical teams [5]. CIs provide filtered information for physicians to improve patient care and help remove the time and expertise barriers to information seeking [6].

Some studies have described the benefits of CI services and their performance. For instance, Zare-Farashbandi et al. considered that the benefits of using CI services for clinical teams included providing necessary information, saving time, reducing costs for health care systems, facilitating publication of treatment results for rare cases, and teaching computer and information retrieval skills [3]. Other studies demonstrated that CIs can improve the information-seeking behavior of clinical professionals and medical students [7, 8]. In a systematic review conducted by Rankin et al., barriers to the use of CIs included the lack of qualified CIs and the need for better definitions of their core competencies [9].

Previous studies have sought to define the competencies that health sciences librarians need [10–12]. The Medical Library Association (MLA) defines professional competencies as basic professional skills and abilities that are observable, are measurable, and can be taught [13]. In particular, MLA identifies six competencies that are required for the professional success of health information professionals: (1) information services, (2) information management, (3) instruction and instructional design, (4) leadership and management, (5) evidence-based practice and research, and (6) health information professionalism [10]. Also, the Australian Library and Information Association defines eight areas of competency for health sciences librarians and information professionals: (1) health environment; (2) reference and research; (3) resources; (4) leadership and management; (5) digital, electronic health, and technology; (6) health literacy and teaching; (7) health research; and (8) professionalism [14].

After Davidoff and Florance introduced the concept of informationists [5], efforts were made to identify competencies and skills that CIs need and to develop training programs. Giuse and Jerome categorized CI competencies into three categories: knowledge, functional abilities, and personal skills [15]. Rankin et al. outlined Giuse and Jerome's competencies with some additional features such as knowledge (e.g., domain, information environment, research design and analysis, technology, organizational, related disciplines, health policy and regulation); functional abilities (e.g., location of information, critical appraisal, information management and organization, project management, knowledge management, education, research, applied informatics, current awareness); and personal attributes (e.g., communication, professionalism, lifelong learning, quality assurance, proactivity, leadership, customer service, entrepreneurialism) [9]. Zare-Farashbandi et al. divided the necessary skills for CIs into three categories: librarianship and informatics skills, medicine-related skills, and general skills [3]. Other studies investigated educational programs for CIs. In particular, Byrd proposed a CI curriculum based on the field of pharmacology [16], Lyon et al. proposed a model for modular education of bioinformaticists [17], and Campbell and Roderer proposed a fellowship program for CIs at Johns Hopkins University [18].

Some studies have described similarities and differences between CIs and clinical medical librarians (CMLs). CIs and CMLs have common tasks: both can participate in clinical rounds, search for evidence for the clinical team, critically appraise the retrieved evidence, and teach information literacy skills to the clinical team [19–21]. However, perhaps the most important differences between CIs and CMLs is that CIs need specialized subject knowledge and should be familiar with medical informatics [5, 22]. Also, whereas the work of CMLs is library based [5], CIs are permanent members of the clinical team [22]. Therefore, CIs may be able to better communicate with clinical team members and meet their information needs.

According to Davidoff and Florance, CIs need knowledge and skills that are gained through formal education [5], which requires identifying their necessary competencies. However, few studies have defined the competencies of CIs. Rather, most existing competencies have been developed for health sciences librarians in general and are only partly suitable for CIs. Therefore, this study aims to identify the specific competencies that CIs require.

Medical library and information science departments in Iranian medical universities were established in 1977 at the master's level [23, 24], followed by the bachelor level in 1986 and doctoral (PhD) level in 2015. Currently, five universities offer medical library and information sciences courses at the bachelor's level, nine universities offer courses at the masters' level, and four universities offer a PhD [25, 26]. Initially, the purpose of these departments was to train qualified librarians for medical libraries. However, with the emergence of new roles for medical librarians, such as CMLs and CIs, these departments have felt the need for change.

In this regard, the Department of Medical Librarianship and Information Sciences of Isfahan University of Medical Sciences launched CI services in 2016 in cooperation with the Department of Internal Medicine. Based on this program, three medical librarianship and information sciences master's students received the necessary additional training in evidence-based databases, advanced searching, evidence-based medicine (EBM), and medical terminology over the course of a year. These librarians, under the guidance of faculty in the Department of Medical Librarianship and Information Science and Department of Internal Medicine, worked as interns for six months in the gastrointestinal ward of Al-Zahra Hospital.

During this period, the librarians enhanced their specialized medical knowledge by doing practical work in the ward. Afterward, they started working professionally as CIs. Their tasks were to search for information at the point of need, teach information retrieval skills to clinical team members, assist clinical team members in conducting research, and prepare case report manuscripts for submission to journals. After evaluating these CI services in the gastrointestinal ward of Al-Zahra Hospital and identifying strengths and weaknesses [3, 8, 27], faculty in the Department of Medical Librarianship and Information Sciences and Department of Internal Medicine decided to identify the competencies that CIs needed with the aim of developing an evidence-based curriculum for CIs.

## METHODS

The authors conducted this study using qualitative content analysis [28]. Our aim was to develop a core competency model for CIs by analyzing the perspectives of major stakeholders, including clinicians, physicians, medical students, CIs, and medical librarians. Because existing competency models were limited, we used the conventional content analysis method, in which researchers do not use preconceived categories but instead allow categories to emerge from the data [28, 29].

Participants were purposefully selected from clinicians who had received CI services for at least two years and medical library professionals who had provided CI services for at least two years in clinical settings at the Al-Zahra Hospital of Isfahan University of Medical Sciences in Iran. A total of twelve clinicians including seven faculty members, three residents, and two medical student interns; seven medical librarians with a master's degree in medical library and information science; and three faculty members in the Department of Medical Library and Information Sciences were selected. Thus, there were a total of twenty-two participants.

We collected data through semi-structured interviews. All participants were interviewed by one of the researchers, who was a PhD student in medical librarianship and information sciences. Interviews lasted twenty to sixty minutes and were typically thirty minutes long. The interviewer obtained informed consent from participants before their interviews (supplemental Appendix A) and recorded the interviews with the permission of the participants. Only one participant did not allow recording; in that case, the interviewer noted important points with the participant's permission. The interview guide included several open-ended questions to allow participants to describe their perceptions and experiences in detail (supplemental Appendix B). At the beginning of the interview, the interviewer asked the participant to describe one day of their work experience related to receiving or providing CI services before proceeding to the other interview questions. If a participant did not understand the meaning of the question, the interviewer rephrased the question or provided additional explanation. Interviews continued until reaching saturation [30].

After each interview, the text of the recorded interview was imported to qualitative data analysis software MAXQDA 10 (VERBI, Berlin, Germany). No demographic information from participants was entered into the software, and a number was assigned to each participant (e.g., P1, P2). We collected and analyzed data simultaneously; each interview was coded and analyzed before the next interview. To identify the unit of analysis, a researcher repeatedly reviewed the texts of the interviews. After deciding that the unit of analysis was a sentence, the researcher read the sentences several times to determine which sentences or phrases had the most meaning to them. These sentences were then analyzed via the constant comparative method. Once the codes were formed inductively, similar codes were merged, and codes with related meanings were grouped to form subcategories. These subcategories were also compared, and those related to the same concept were grouped to form categories.

We ensured the credibility, transferability, dependability, and confirmability of the findings by having an external reviewer recode the text of the interviews. There was an average 83% agreement on categories and subcategories extracted by the original researcher and external reviewer. Cases of disagreement were discussed jointly to reach final agreement. Also, when contacting 5 participants, the interviewer provided them with a summary of extracted themes along with example quotations, and participants were asked to confirm whether the themes and quotations were clear and appropriate. There was 75%–92% agreement among these 5 participants.

## RESULTS

We interviewed twenty-two participants, consisting of clinicians who had received CI services and medical library professionals who had provided CI services.

Qualitative content analysis led to the identification of six main categories and twenty-five subcategories (Table 1). Whereas clinicians placed more importance on research and domain knowledge categories, librarians placed more importance on the information services category.

**Table 1:** Categories and subcategories of clinical informationist (CI) competencies

Main categories	Subcategories	Percent of respondents		
		Clinicians	Library professionals	Total
Communication	Leadership skills	42%	30%	36%
	Group work skills	67%	50%	59%
	Critical thinking skills	50%	80%	64%
	Professionalism	30%	70%	50%
Research	Understanding and use of evidence-based medicine (EBM)	58%	90%	73%
	Medical research methods	83%	80%	82%
	Critical appraisal skills	50%	50%	50%
	Scholarly communication	67%	50%	59%
Education and training	Design and implementation of face-to-face and online training programs	42%	80%	59%
	Use of the latest educational approaches and methods	42%	70%	55%
	Design and preparation of printed and nonprinted educational resources	83%	80%	82%
Domain knowledge	Medical terms and acronyms	100%	80%	91%
	Basic and specialized medical and clinical concepts	100%	70%	86%
	Medical tests	33%	60%	46%
	Patient records	50%	50%	50%
	Health care environment and policies	50%	80%	64%
Information services	Assessment of information needs	42%	90%	64%
	Advanced search	67%	100%	82%
	Documentation	33%	70%	50%
	Information organization and management	42%	80%	59%
	Information visualization	58%	60%	59%
	Current awareness services (CAS) and selective dissemination of information (SDI)	50%	90%	68%
Technology	Use of the latest technologies to provide information services, education and training, and research	58%	100%	77%
	Communication technologies	67%	80%	73%
	Health information systems	58%	70%	64%

### Communication

#### Leadership skills.

Given that CIs work in clinical teams, they need to have strong leadership skills to serve as influential members of the team. Over one-third of participants believed that CIs should have leadership skills.

If he cannot plan the work himself, it is useless, there are lots of patients, the crowded setting, student education, and…He has to plan and direct the work related to CIs by himself. (P8: clinician)

#### Group work skills.

As CIs are members of clinical teams, having teamwork skills is a prerequisite for their role. Over half of participants considered group work skills as necessary for CIs.

Everyone has a duty in the clinical team; the CI also has responsibilities in this team…[The] CI should know how to treat each member of the team. (P5: librarian)

#### Critical thinking skills.

Most library professionals and half of clinicians believed that CIs should have critical and creative thinking skills.

When I first joined the clinical team, I was confronted with a more different world than I had imagined. I had to learn many things. I needed to decide at the moment. I think strategic critical thinking is the best tool for me as a CI. (P5: librarian)

#### Professionalism.

As CIs are relatively new specialists in the field of medical library and information science, CIs entering clinical teams should help clinical and medical professionals better understand the CI profession through their professional behavior.

A CI must be able to demonstrate effectively their skills to physicians so that they can trust the librarian and ask their information needs from the CI. (P9: librarian)

### Research

#### Understanding and use of evidence-based medicine (EBM).

Most participants emphasized that CIs should be familiar with the EBM process.

Another case is EBM and evidence levels in clinical medicine. The CI should have a good command of EBM such that the doctor feels they should see a librarian even before they think about their need. (P5: librarian)

#### Medical research methods.

Most participants stated that CIs should be familiar with medical research methods and be able to design medical and clinical studies. This type of knowledge and skill set received a high degree of agreement between library professionals and clinicians.

If there is someone familiar with medical research, they can help a lot. For example, when I am writing a proposal, an informed librarian is very helpful…Well, it can [be] very useful with writing methodology. (P1: clinician)

#### Critical appraisal skills.

Because CIs should provide quality information and evidence to clinicians for decision making, they must have critical appraisal skills. Half of participants emphasized these skills.

The CI searches for information and gives me the same search results; maybe this is not good for me. He should separate the best of them for me. (P8: clinician)

#### Scholarly communication.

CIs should be familiar with various methods of disseminating the results of medical research and be able to publish and share those results in the most effective manner. Over half of participants described scientific communication skills as a basic skill for CIs.

We are now designing and submitting case reports of the internal department with the help of CIs. We had not even thought about it, the CIs gave us the idea to share the case reports. (P12: clinician)

### Education and training

#### Design and implementation of face-to-face and online training programs.

CIs can play an important role in training clinical team members, patients, and their families. Library professionals emphasized the role of CIs in designing training programs more than clinicians.

We set up search skills training workshops for medical students. Students welcome so much…these workshops and even residents asked for the search-training workshop. We even provided in-person training via telegram for those who could not attend the workshop. (P2: librarian)

#### Use of the latest educational approaches and methods.

To increase the effectiveness of education and training, CIs should use the latest teaching methods, including student-centered training.

In the first session of the workshop that I had set up for them, one of the medical students claimed that these workshops were not useful for them and that only Medscape could help them. I told him it is OK, you teach the first session. He trained [about] Medscape. When I started, they knew Medscape is not the only database suitable for them. They did not know other databases at all. (P9: librarian)

#### Design and preparation of printed and nonprinted educational resources.

CIs should provide appropriate educational resources in printed and nonprinted formats for the clinical team and patients. Most participants mentioned this role, with a high degree of agreement between library professionals and clinicians.

The CI should provide information in the form of infographics using Microsoft PowerPoint, to provide training pamphlets for patient education, or write a medical text for the use of patients in a simple form. (P3: librarian)

### Domain knowledge

#### Medical terms and acronyms.

All clinicians and most library professionals stated that CIs should be familiar with medical terms and acronyms.

I think the CI should know at least the basic medical acronyms. For example, if he/she is going to work in the [ear, nose, throat] ENT section, he/she should at least know the acronyms of the ENT section. (P5: clinician)

#### Basic and specialized medical and clinical concepts.

CIs should be able to understand and use medical knowledge, which can help them more effectively search for evidence. All clinicians and most library professionals stated that CIs should know medical concepts such as methods of diagnosis and treatment of diseases, basic and specialized medical concepts, and principles of pharmacology.

The CI should know some of the primary diseases, such as [chronic obstructive pulmonary disease] COPD or asthma; not the exact definition, but at least the abbreviations of lung diseases. (P3: clinician)

#### Medical tests.

CIs should be familiar with common and specialized laboratory tests in their clinical team's field.

We need to know some laboratory tests. I consider it a weakness that we, like CI, cannot diagnose laboratory tests, testing analysis is a doctor's job, but we must have at least some information about them. (P1: librarian)

#### Patient records.

CIs should be familiar with patient medical records and how to use them.

Our colleague never sees the patient first; first, she sees the patient's medical record because she has to search and this is the part where the CI can help much. (P7: clinician)

#### Health care environment and policies.

CIs should be able to understand clinical policies and environments, including hospital wards, national and international health organizations, health care staff duties, health care personnel dress codes, health care personnel hierarchy, health care policies, and standards.

Familiarity with the clinical environment can be very helpful…In the healthcare environment; everyone has his or her clothes. We do not learn these. (P6: librarian)

### Information services

#### Assessment of information needs.

CIs should be able to identify the information needs of clinical team members through a variety of approaches, such as performing reference interviews and assessing information behaviors. Over half of participants mentioned information needs assessment as a basic skill for CIs.

I need to know what my user wants. The clinical specialist wants one thing and the medical student wants another; and these are different. (P2: librarian)

#### Advanced search.

CIs should be able to perform advanced and complex searches in clinical and evidence-based databases. Most participants emphasized the advanced search skills of CIs.

We often do simple searches. Occasionally, we may see some rare cases that no one has ever encountered, and we do not get much when we search. The CI should be able to find accurately rare case reports for us. (P3: clinician)

#### Documentation.

A CI should be able to record, store, and document valuable information using methods such as database creation.

Clinicians use rare cases in their lessons, but they do not share. You can collect these and create a database that has an educational aspect for others. A CI can do this. (P7: librarian)

#### Information organization and management.

Information organization and management were CI skills mentioned by over half of participants.

Another thing is to help me summarize texts that are very broad and give them to me in the form of tables and diagrams. (P9: clinician)

#### Information visualization.

CIs should be able to visualize information for clinicians and patients through infographics, videos, and other formats. Both clinician and library professionals noted these skills.

For example, the doctor says I want to prepare a video case study of my patient. Help me make a video case. You have to prepare the text and help make the clip. (P9: librarian)

#### Current awareness services and selected dissemination of information.

One competency highlighted by participants was the provision of current awareness services (CAS) and selected dissemination of information (SDI).

Another thing the librarian can do for us is creating a capsule summary. For example, the CI may tell the doctor that a new issue of a journal has been published and ask the doctor if [they] would like the contents of the journal to be summarized for them. (P6: clinician)

### Technology

#### Use of the latest technologies to provide information services, education and training, and research.

A CI should be able to use the latest technologies to provide information services, train the clinical team and patients, and manage research data. All participants mentioned these skills.

Devices and technology are growing so much that students who graduate after 2 years will see that the technology they have been trained with is very different from the technology they have now. (P5: librarian)

#### Communication technologies.

Clinicians have many tasks, especially in educational hospitals, including caring for patients, educating students and residents, and performing research. Clinicians sometimes lack time and prefer virtual or asynchronous services of CIs. Therefore, CIs should be able to use the latest technologies to communicate effectively and provide the best services remotely.

It is great that the CI can offer me services via the internet, social networks, and so on. (P8: clinician)

#### Health information systems.

To manage health information, CIs must be familiar with health information systems and be able to use or even develop these systems.

We have some case reports that need to be stored somewhere so that they can be used to create a database for us. CIs can cooperate in designing and entering the data. (P2: clinician)

## DISCUSSION

We found that clinicians placed the most emphasis on research and domain knowledge competencies. Thus, CIs must be sufficiently familiar with medical research and have sufficient mastery of medical knowledge, which will help them perform more accurate searches for evidence. Other studies also addressed the importance of familiarity with medical research and domain knowledge in empowering CIs [7, 17]. In contrast to clinician participants, librarian participants were more concerned with the information services competency. Perhaps the reason why clinicians paid less attention to information services was that they were not fully familiar with all of the services that a CI can provide. Therefore, CIs may need to market their services in a more effective manner.

**Figure 1:**
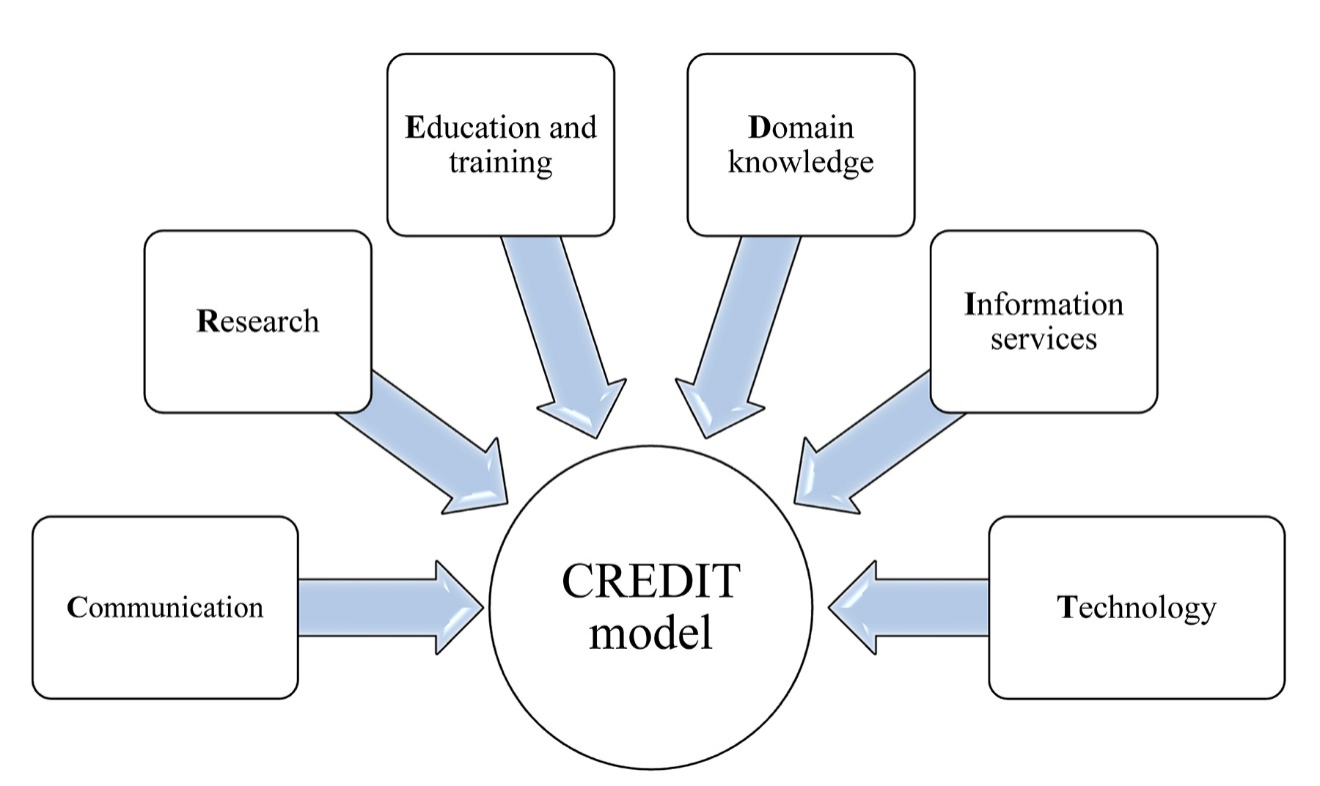
CREDIT core competency model for clinical informationists (CIs)

The ultimate mission of CIs is to improve health care services. CI services reduce health care costs [3, 27], improve evidence-based practice [7], and improve the quality of patients' treatment [31]. However, as the role of CIs is highly specialized, they must have competencies in specific areas. To identify these competencies, we analyzed the opinions of major stakeholders in CI services. The results of this analysis led us to develop a CREDIT core competency model for CIs that includes six competencies that fully cover the skills required of CIs who are members of clinical teams: communication, research, education and training, domain knowledge, information services, and technology (Figure 1).

Whereas CIs are expected to be highly capable in each of these six competencies, other types of health sciences librarians and information professionals are not expected to have all of these competencies to the same desired level [10, 14]. However, as CIs and CMLs provide similar services, the CREDIT model can also be useful for CMLs, especially for developing and evaluating educational and training programs.

The first competency, which was highly emphasized by both librarians and clinicians and, thus, may be the “golden key” for CIs, is communication. Strong communication skills can greatly ensure the success of CIs [3], and Giuse and Jerome consider communication a personal competence for CIs [15]. One of the findings of this study was that clinicians, despite using CI services, still focused on CIs' searching skills, perhaps due to their unfamiliarity with all of the duties of CIs. Thus, CIs should make more effort to introduce this new profession to potential stakeholders. One way to market CI services is to provide services with the highest perceived quality, in which case clinicians who need and receive these services will tend to continue using those services.

Research is the second competency that CIs need in order to support evidence-based practice. Giuse and Jerome cite research as a functional competence [15], and Zare-Farashbandi et al. suggest that mastery of research methods should be a part of the general skills of CIs [3]. Studies developing a curriculum for CIs also emphasize the need for research skills [17, 18]. Therefore, CIs should be able to understand and use evidence-based practice processes. They should be able to design a variety of clinical research studies, develop and assess methodologies, and publish and share the results of clinical studies.

The third competency of CIs is education and training. Zare-Farashbandi et al. consider teaching databases to members of the clinical team as a skill for CIs [3]. Giuse and Jerome consider education to be a functional competency of CIs [15]. Tahmasebi et al., Marshall and Neufeld, McGowan, and Rosenberg et al. also mention the educational role of CIs [8, 32–34]. This educational role of CIs can be considered in two ways: one is teaching information literacy to clinicians, and the other is teaching health literacy to patients and their families. Toward this end, CIs should be able to teach information literacy skills to strengthen clinicians' evidence-based practice and work with other health care professionals to teach health literacy to patients and their families.

The fourth competency of CIs is domain knowledge. Because they work in a clinical setting, they should be able to understand the language of experts in their field. What sets CIs apart from other health sciences librarian roles is specialized medical domain knowledge [21]. Davidoff and Florance believed that CIs should have strong specialized medical knowledge [5]. Other studies suggest that medical domain knowledge is essential for CIs [3, 15, 16, 18]; however, domain knowledge is not mentioned in the MLA competencies [10]. Thus, this study establishes medical domain knowledge as a fundamental competence for CIs. Furthermore, CIs must have general medical knowledge as well as specialized knowledge in the field in which they work. For example, if they work in the cardiology department, they should be familiar with its terms, acronyms, diseases, medications, and diagnostic and treatment methods.

The fifth competency that CIs require is information management and services, which is also emphasized by other studies on CIs [3, 5, 15]. CIs should be able to assess the information needs of clinicians, use advanced search skills, manage and organize the retrieved information, and provide clinicians with information through visualizations and summaries. The results of this study show that clinicians prefer to perform simple searches by themselves and seek help from a CI for searching complex and rare cases. Therefore, CIs should be able to search for rare cases at the point of need and have the skills to manage and organize medical and clinical information.

The sixth competency required by CIs is technology. Although MLA does not mention technology as a separate competency [10], we consider it a separate competency to give it more attention for the development of curriculums. Technology skills for CIs are emphasized by Davidoff and Florance [5] as well as other researchers [3, 15]. Given that technologies are constantly evolving, our study points to the need to focus on new technologies. CIs should be able to use the latest technologies to provide their services, which requires lifelong learning.

CIs must have specialized competencies to be able to fulfill their roles on clinical teams and, thereby, promote health services and improve patient care. As such, the CREDIT model can be used as criteria for evaluating the performance of CIs. Moreover, planners and policymakers in the field of health sciences librarianship education can employ this model to develop and assess educational programs and curriculums. This study is part of a larger study on curriculum development for CIs. The interdisciplinary and specialized nature of CIs and the connection of this specialty to clinical fields highlights the need for training CIs [9]. Therefore, in the next phase of this research, we will develop an appropriate curriculum for CIs based on these core competencies.

## Data Availability

Data associated with this article are available in Harvard Dataverse at https://dataverse.harvard.edu/dataset.xhtml?persistentId=doi:10.7910/DVN/AZCQHG.

## SUPPLEMENTAL FILES

**Appendix A:** Consent form for interview

**Appendix B:** Semi-structured interview guide
