# Supplementary material for: A core competency model for clinical informationists
Source: J Med Libr Assoc. 2021 Jan 1;109(1):33–43. doi: 10.5195/jmla.2021.1065 (PMC7772977; doi:10.5195/jmla.2021.1065)
Supplement: Supplementary file 1 — Appendix A: Consent form for interview [file jmla-109-1-33-s01.pdf]

Journal of the Medical Library Association

Supplemental content to
J Med Libr Assoc. Jan;109(1):dx.doi.org/10.5195/jmla.2021.1065
www.jmla.mlanet.org
© Hashemian, Zare-Farashbandi, Yamani, Rahimi, Adibi 2021

A core competency model for clinical informationists

Mohammadreza Hashemian; Firoozeh Zare-Farashbandi; Nikoo Yamani; Alireza Rahimi; Peyman Adibi

APPENDIX A

Consent form for interview

Thank you for participating in this interview. If you agree to participate, please read and sign the form below:

I have had the purpose and nature of the study explained to me in writing, and I have had the opportunity to ask questions about the study. I understand that I can withdraw at any time or refuse to answer any question without any consequences of any kind. I understand that my response will be kept strictly confidential. I understand that in any report on the results of this research my identity will remain anonymous. This will be done by changing my name and disguising any details of my interview that may reveal my identity or the identity of the people I speak about. I agree to my interview being audio-recorded. I understand that the audio recording made of this interview will be used only for analysis.

I agree to take part in this interview.

Name of participant
Date
Signature

Name of researcher
Date
Signature
