# Supplementary material for: A core competency model for clinical informationists
Source: J Med Libr Assoc. 2021 Jan 1;109(1):33–43. doi: 10.5195/jmla.2021.1065 (PMC7772977; doi:10.5195/jmla.2021.1065)
Supplement: Supplementary file 2 — Appendix B: Semi-structured interview guide [file jmla-109-1-33-s02.pdf]

Journal of the Medical Library Association

Supplemental content to
J Med Libr Assoc. Jan;109(1):dx.doi.org/10.5195/jmla.2021.1065
www.jmla.mlanet.org
© Hashemian, Zare-Farashbandi, Yamani, Rahimi, Adibi 2021

A core competency model for clinical informationists

Mohammadreza Hashemian; Firoozeh Zare-Farashbandi; Nikoo Yamani; Alireza Rahimi; Peyman Adibi

APPENDIX B

Semi-structured interview guide

Hello and thank you for participating in this interview. My name is ___________________ and I am interviewing to identify the core competency for clinical informationist. Written consent was obtained from you. The interview takes about fifteen to sixty minutes. Let's start.

1. Please talk about your experience with clinical informationist services. Describe a working day.
2. What problems have you encountered in accessing information?
3. What services do you expect the clinical informationist to provide to you?
4. What competencies and skills do you think a clinical informationist should have?

Thank you very much.
